# Warmer waters masculinize wild populations of a fish with temperature-dependent sex determination

**DOI:** 10.1038/s41598-019-42944-x

**Published:** 2019-04-25

**Authors:** J. L. Honeycutt, C. A. Deck, S. C. Miller, M. E. Severance, E. B. Atkins, J. A. Luckenbach, J. A. Buckel, H. V. Daniels, J. A. Rice, R. J. Borski, J. Godwin

**Affiliations:** 1 North Carolina State University, Department of Biological Sciences, Raleigh, NC 27695 USA; 2 North Carolina State University, Department of Applied Ecology, Raleigh, NC 27695 USA; 3 Environmental and Fisheries Sciences Division, Northwest Fisheries Science Center, National Marine Fisheries Service, National Oceanic and Atmospheric Administration, 2725 Montlake Blvd E, Seattle, WA 98112 USA

**Keywords:** Climate-change ecology, Ecophysiology, Climate-change ecology, Climate-change impacts

## Abstract

Southern flounder (*Paralichthys lethostigma*) exhibit environmental sex determination (ESD), where environmental factors can influence phenotypic sex during early juvenile development but only in the presumed XX female genotype. Warm and cold temperatures masculinize fish with mid-range conditions producing at most 50% females. Due to sexually dimorphic growth, southern flounder fisheries are dependent upon larger females. Wild populations could be at risk of masculinization from ESD due to globally increasing water temperatures. We evaluated the effects of habitat and temperature on wild populations of juvenile southern flounder in North Carolina, USA. While northern habitats averaged temperatures near 23 °C and produced the greatest proportion of females, more southerly habitats exhibited warmer temperatures (>27 °C) and consistently produced male-biased sex ratios (up to 94% male). Rearing flounder in the laboratory under temperature regimes mimicking those of natural habitats recapitulated sex ratio differences observed across the wild populations, providing strong evidence that temperature is a key factor influencing sex ratios in nursery habitats. These studies provide evidence of habitat conditions interacting with ESD to affect a key demographic parameter in an economically important fishery. The temperature ranges that yield male-biased sex ratios are within the scope of predicted increases in ocean temperature under climate change.

## Introduction

Fish are strongly influenced by climate variability with regards to reproduction, productivity, food availability, and recruitment^1^. Global temperatures are on the rise and sea surface temperatures have been increasing at an average rate of 0.18 °C per decade, with coastal waters having a greater rate of increase than open oceans^2^. Some models have predicted that global temperatures may rise between 2.0–4.9 °C by 2100 with only a 1% chance the increase will be below 2.0 °C^3^. This raises concerns about impacts of warming habitats on marine ecosystems and resources. Loss of suitable habitat, possible shifts in distribution and larval dispersal, and changes to larval development time have been suggested^1,2,4,5^. Northward shifts in distribution have been found for many species on the North American Atlantic coast^6,7^, including juvenile bull sharks from the same geographic region as the current study^8^. Importantly, these shifts are predicted to continue in the future^9^.

Many species of fish and reptiles exhibit temperature-dependent sex determination (TSD), a phenomenon that occurs when an organism’s sex can be permanently influenced by the temperature in their surrounding environment, typically during a critical period of early development^5,10,11^. When factors in the environment including temperature affect an organism’s sex, it is termed environmental sex determination (ESD)^12,13^. Teleost fishes display a great deal of sexual plasticity and a wide range of sex determination patterns including those in which sex is determined by cues from the physical and social environments^14–18^. There are variations of TSD/ESD where animals can exhibit true TSD/ESD, or genetic sex determination (GSD) with temperature effects (GSD + TE), however, in either case the animal’s phenotypic sex is dictated by the environment^5,19^. Understanding the consequences of climate change on those species that exhibit TSD and are sensitive to temperature in their environment is critical^5,20^. Studies of the potential impacts of climate change on reptiles that exhibit TSD suggest that even increases in mean temperatures of less than 2 °C could severely skew sex ratios^21^. Indeed, effects of this type have been recently described in green sea turtles on the Great Barrier Reef where warming temperatures were associated with biased sex ratios^22^.

Paralichthid flounder are a genus of benthic flatfish that supports major commercial and recreational fisheries throughout the world. Southern flounder (*Paralichthys lethostigma*) are distributed in the southeastern United States, with populations in the Gulf of Mexico and along the Atlantic coast in North Carolina (NC), near the northern edge of the species range. Adult flounder are batch spawners that migrate out from estuaries to coastal waters to spawn during late fall to early winter^23^. The fertilized eggs are buoyant and will float for two to three days until hatching. After one to two months of development in offshore waters, the larvae undergo metamorphosis before moving into rivers and settling into estuarine nursery habitats^24,25^. Juvenile southern flounder habitats typically include estuarine waters with aquatic vegetation, shells, and mud bottoms along saltmarsh edges^26,27^. Southern flounder exhibit sexually dimorphic growth, with females growing larger and faster relative to males^28^. Due to the use of a minimum size limit as a management strategy, the southern flounder fishery is heavily dependent on females, with males rarely growing to a harvestable size^28,29^. A reduction in the proportion of females translates directly into a reduction in recruitment to the fishery. Based on survey data, there are declining trends in juvenile and adult abundances, and southern flounder are under “concerned” status^29–32^. Recent assessments in NC have indicated that southern flounder are overfished, and the species was updated to “near threatened” on the International Union for Conservation of Nature (IUCN) Red List in 2015^31,33^.

Southern flounder and other Paralichthids exhibit TSD/ESD and appear to use a combination of genotypic and environmentally determined systems, where an animal with an XY genotype is a genetic male and XX is female. Individuals of XY genotype will develop as phenotypic males regardless of environmental conditions, however, XX genetic females may differentiate into phenotypic males (XX males) based on the environmental parameters to which they are exposed to^34–37^. When juveniles are reared in cooler or warmer water temperatures (18 °C and 28 °C^35^) or blue background color in tanks^37^ during a critical early developmental stage (~35 to 65 mm total length, TL^38^), genetic females develop as males leading to masculinized sex ratios. Thus, the maximum proportion of females that can occur is approximately 50%, with male-biased sex ratios possible if temperature or other factors lead to masculinization of XX females. To date, no studies have investigated the effects of environment on juvenile sex ratios of southern flounder populations or any Paralichthids in the wild. If these fish are exposed to unfavorable conditions during the sex determination window, then wild stocks could be at risk of masculinization which would negatively impact an already declining female-dependent fishery. It is currently unknown if wild juvenile southern flounder exhibit annual or geographic variation in sex ratios. When temperature is held constant in tanks, southern flounder exhibit sex reversal to males at both 18 °C and 28 °C, while 23 °C is the optimal temperature for female development^35^. Nevertheless, water temperature is a dynamic variable and does not remain constant in natural environments, instead fluctuating both daily and annually. It has been suggested that studies where temperatures are held constant may not be ideal for modeling impacts of natural environments^39^.

The sex-determining window for southern flounder is estimated to occur between 35 and 65 mm TL^38^, while the fish are still in their nursery habitats. While gonadal sex differentiation generally occurs between 75 and 120 mm TL, fish cannot be accurately sexed via histological or macroscopic methods until reaching at least 120 mm TL^35^. It has therefore been challenging to determine sex ratios of juvenile populations by traditional methods. Because management agencies cannot assess the sex of young juveniles, most fishery models assume a 1:1 juvenile sex ratio^29^. Despite considerable study of sex determination in Paralichthid flounders, to date no Y-chromosomal marker has been described for this family of fishes. To enable characterization of nursery habitat influences on sex determination, we developed and validated a molecular biomarker technique based on gonadal expression of genes involved in sexual differentiation that allows us to sex individuals at much smaller sizes^37^. Using this approach, young southern flounder can be captured from their juvenile habitats and sexed before reaching a body size when they emigrate from nursery areas and potentially mix with other juveniles in deeper estuarine waters^26^. Previous studies show that post-metamorphic juvenile southern flounder settle into nursery habitats and remain around these estuarine environments for the first year of life with relatively small spatial movements^32,40^. Because of this aspect of the flounder life history and the ability to reliably sex flounder using gonadal biomarkers prior to moving out of nursery habitats, we can evaluate effects of habitat on sex ratios of flounder populations.

Here, we examine the sex ratios of juvenile southern flounder populations across several years and in a number of nursery locations along the coast of NC, USA that show consistent differences in water temperatures. Additionally, we tested the hypothesis that patterns of temperature variation observed in NC nursery habitats that produce male-biased juvenile sex ratios are sufficient (by themselves) to generate these skewed sex ratios. By mimicking these temperature profiles under controlled laboratory conditions while other variables were held constant, we directly examined the effects of fluctuating temperature on sex determination.

## Results

### Temperature of Flounder Nursery Habitats

**Figure 1:**
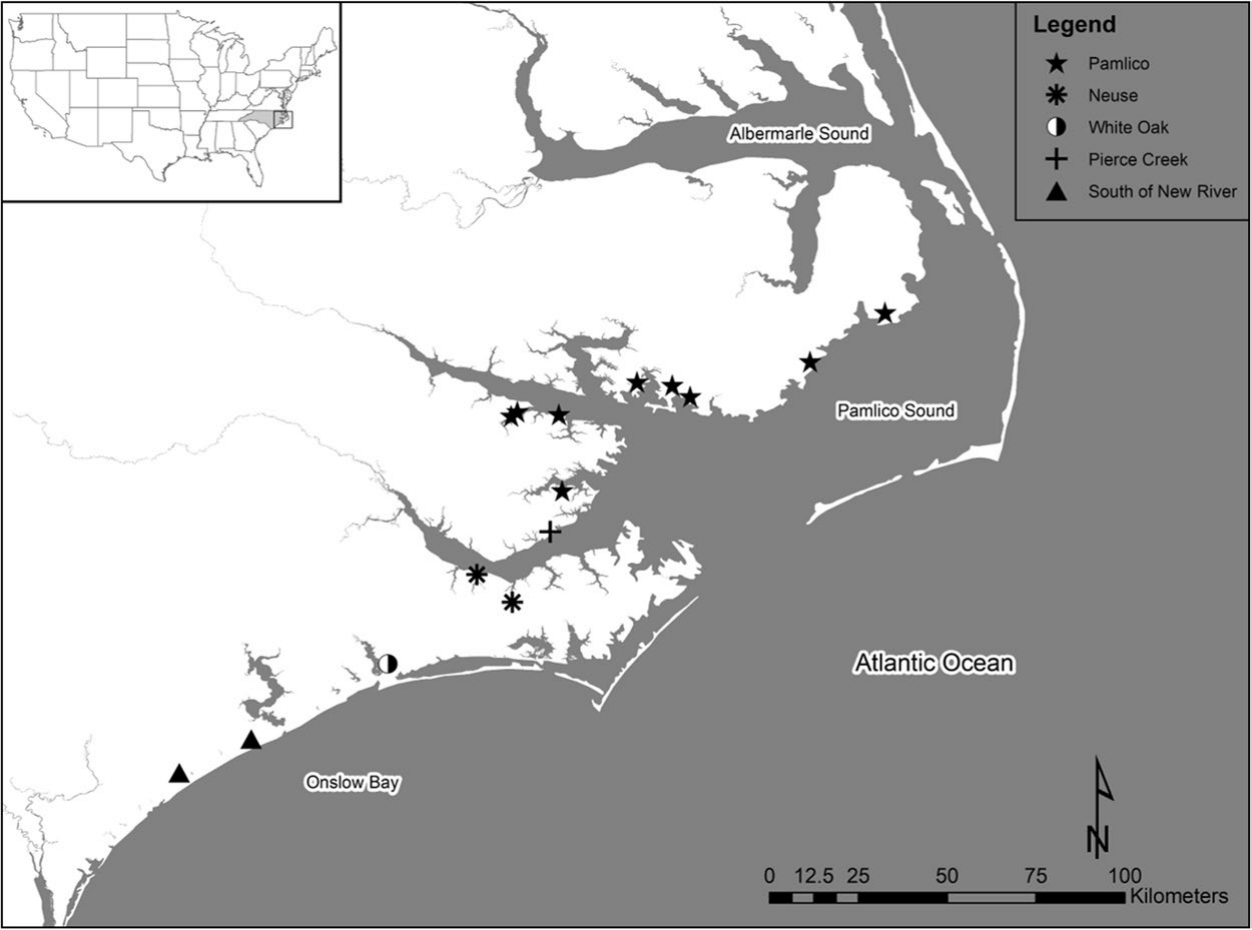
Map showing the location of temperature probes deployed in North Carolina juvenile southern flounder nursery habitats from 2014 to 2017. Markers indicate the location of a single temperature logger (many additional sampling sites where fish were captured are not shown). Sites were grouped into three regions: (1) Pamlico (stars; northern), (2) Neuse (asterisks; intermediate), (3) South of the New River (triangles; southern). There were temperature loggers at two additional sites, one in Pierce Creek (plus sign) and one in the White Oak River (half circle), where temperature was recorded but the site was not included in any of the three regions. Map was created with Environmental Systems Research Institute, ArcGIS Desktop 10.3.1. (ESRI 2018: Redlands, CA, USA, www.esri.com).

**Figure 2:**
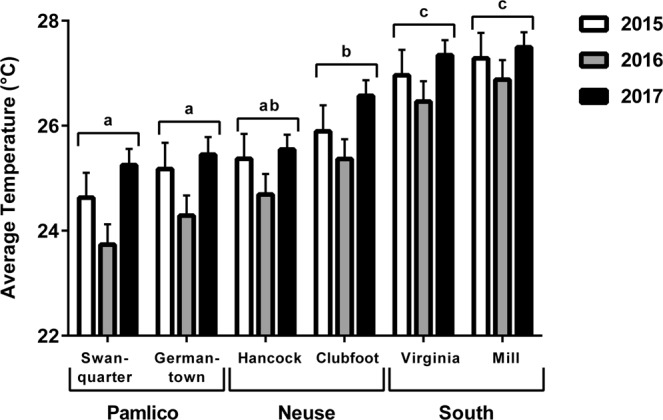
Average annual water temperature from representative juvenile southern flounder habitats in years 2015, 2016, and 2017 (April 16 to June 30). Temperature was recorded every 2 hours and readings were averaged for each 24-hour period. Letters indicate differences among habitats over the three years taken together (ANOVA, *P* < 0.0001). With all habitats combined the effect of year was significant for 2016 (ANOVA, *P < 0.0001).

Data were grouped into three sampling regions that span a biogeographic break resulting from the meeting of the warmer Gulf Stream and the cooler Labrador Current (Fig. 1): (1) the Pamlico region, the most northern of our sampling locations including several sites in the Pamlico River and tributaries, (2) the Neuse River; an intermediate region including three sites, and (3) South of the New River, the most southern region, primarily sampled from Mill Creek and then supplemented with fish from two other nearby creeks. Temperature loggers were placed in representative NC juvenile southern flounder nursery habitats across four years: 2014 (11 probes), 2015 (13 probes), 2016 (14 probes) and 2017 (13 probes) (Fig. 1). Temperature readings fluctuated daily and annually as anticipated (see Supplemental Figs 1 and 2 for annual temperature profiles). The average daily temperature from all probes in the three regions between April 16 and June 30 in each sampling year were: Pamlico: 2014 = 24.8 °C, 2015 = 25.6 °C, 2016 = 24.5 °C, 2017 = 25.7 °C; Neuse: 2015 = 25.6 °C, 2016 = 25.0 °C, 2017 = 26.1 °C; and South of the New River: 2015 = 27.1 °C, 2016 = 26.7 °C, 2017 = 27.4 °C. The average maximum difference in temperature recorded between habitats was 2.8 °C in 2014, 3.7 °C in 2015 and 2016, and 3.3 °C in 2017. Across years the habitats that consistently recorded the warmest temperatures were in the region South of the New River, i.e. Mill and Virginia Creeks. Similarly, when average annual water temperature from two representative creeks from each region (Virginia and Mill Creeks in the South, Clubfoot and Hancock in the Neuse River, Swanquarter and Germantown in the Pamlico) were analyzed from April 16 to June 30 in years 2015 through 2017, areas South of the New River experienced the warmest temperatures, the Neuse River experienced intermediate temperatures, and the Pamlico experienced the coolest temperatures (Fig. 2, *P* < 0.0001). With all habitats combined, the effect of year shows the temperature was cooler in 2016 than in 2015 or 2017 (Fig. 2, *P* < 0.0001). In 2014, temperature loggers were not deployed early enough in all habitats to be included in analyses.

### Flounder HSI and Total Length

**Figure 3:**
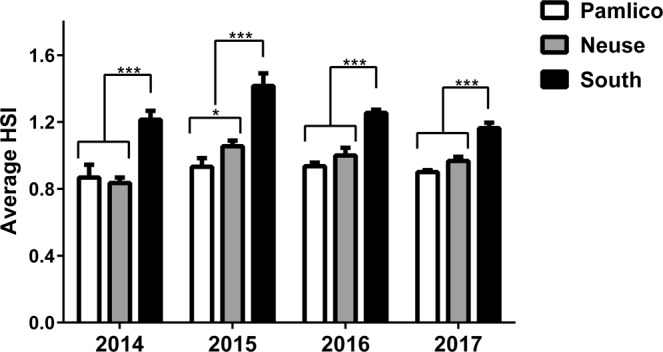
Average hepatosomatic index (HSI) for juvenile southern flounder across three sampling regions; Pamlico (northern), Neuse (intermediate), and South, over four years from 2014 to 2017 (*P = 0.02, ***P ≤ 0.0003; ANOVA). HSI = (Liver weight/Total body weight) × 100.

Hepatosomatic index (HSI) is a useful indicator of fish health and overall energy stores reflective of recent food consumption^41,42^. It is calculated as the weight of liver, a primary storage site for glycogen and fats, as a percentage of total body weight of an individual. Values were calculated for individual samples and then tabulated into regional HSI for each year. The regional HSI values ranged from (expressed as mean ± standard error; SEM): Pamlico: 2014 = 0.87 ± 0.08, 2015 = 0.93 ± 0.05, 2016 = 0.94 ± 0.02, 2017 = 0.90 ± 0.01; Neuse: 2014 = 0.83 ± 0.03, 2015 = 1.06 ± 0.03, 2016 = 1.00 ± 0.05, 2017 = 0.97 ± 0.02; South: 2014 = 1.21 ± 0.05, 2015 = 1.42 ± 0.08, 2016 = 1.25 ± 0.02, 2017 = 1.16 ± 0.03. Over all sampling years, the region South of the New River steadily produced flounder with the greatest average HSI (Fig. 3, *P* < 0.0001). In addition, the flounder collected over all sampling years from South of the New River were consistently the smallest fish when compared to flounder sampled in other locations over the same sampling months (Table 1; also see Supplemental Fig. 3 for individual samples). They were on average 11.2 mm TL smaller than flounder collected in the Pamlico or Neuse Rivers over all years, with a maximum difference of 19.2 mm TL smaller than flounder from the Pamlico River in May of 2016 and a minimum difference of 0.6 mm TL smaller than the fish captured in the Neuse River in June of 2017. We observed two distinct size groupings of flounder in two sampling years south of the New River in Mill Creek. In May 2016, of the 57 southern flounder captured, 51 fish (37 males, 1 female, and 12 undetermined) were on average 50.1 mm TL, five fish were on average 120.0 mm TL (5 females) and one male was 235 mm TL. In June 2016, of the 49 total southern flounder captured, 39 fish (34 males, 2 females, and 3 undetermined) were on average 56.1 mm TL, and 10 fish (2 males and 8 females) that were on average 145.6 mm TL. Similarly, in Virginia Creek, also South of the New River, in June of 2016 there were eight fish that were on average 50.8 mm TL (7 males and 1 female), and two fish (2 females) that were on average 146.0 mm TL. In June of 2017 there were 49 fish captured in Mill Creek that were on average 69.7 mm TL (48 males and 1 female), four fish that were on average 146.0 mm TL (2 males and 2 females), and two fish (1 male and 1 undetermined) that were on average 254.5 mm TL. Fish >200 mm are likely not young-of-the-year (YOY).

**Table 1:** Average length (mm TL) ± SEM, range of mm TL, date of sampling, number of juvenile southern flounder collected from 2014 to 2017 in three different regions: Pamlico (north), Neuse (intermediate), and South.

Region	Date	#Fish	Avg. mm TL	Range mm TL
Pamlico	June, 2014	41	90.7 ± 4.63	63–230
Neuse	June, 2014	54	84.7 ± 2.05	58–118
Neuse	July, 2014	41	107.0 ± 2.13	77–135
South	June, 2014	18	75.9 ± 5.93	50–170
Pamlico	May, 2015	85	68.6 ± 1.63	50–123
Neuse	May, 2015	80	64.5 ± 2.39	50–240
Neuse	June, 2015	73	81.1 ± 1.42	58–115
South	June, 2015	15	68.0 ± 3.84	54–114
Pamlico	May, 2016	59	79.5 ± 4.29	51–240
Pamlico	June, 2016	58	81.2 ± 2.45	52–173
Pamlico	July, 2016	4	98.5 ± 4.72	92–112
Neuse	May, 2016	62	71.1 ± 4.85	54–262
Neuse	June, 2016	40	91.2 ± 2.87	51–122
Neuse	July, 2016	39	84.3 ± 1.45	69–107
South	May, 2016	57	60.3 ± 4.33	40–235
South	June, 2016	59	73.6 ± 5.30	40–194
Pamlico	May, 2017	91	76.3 ± 2.62	51–225
Pamlico	June, 2017	66	85.2 ± 1.61	65–116
Pamlico	July, 2017	32	86.6 ± 1.95	66–112
Neuse	May, 2017	32	67.8 ± 3.49	49–160
Neuse	June, 2017	85	82.7 ± 1.64	63–135
South	May, 2017	11	58.4 ± 3.56	46–89
South	June, 2017	54	82.2 ± 5.48	55–268

### Sex Ratios of Wild Southern Flounder

**Figure 4:**
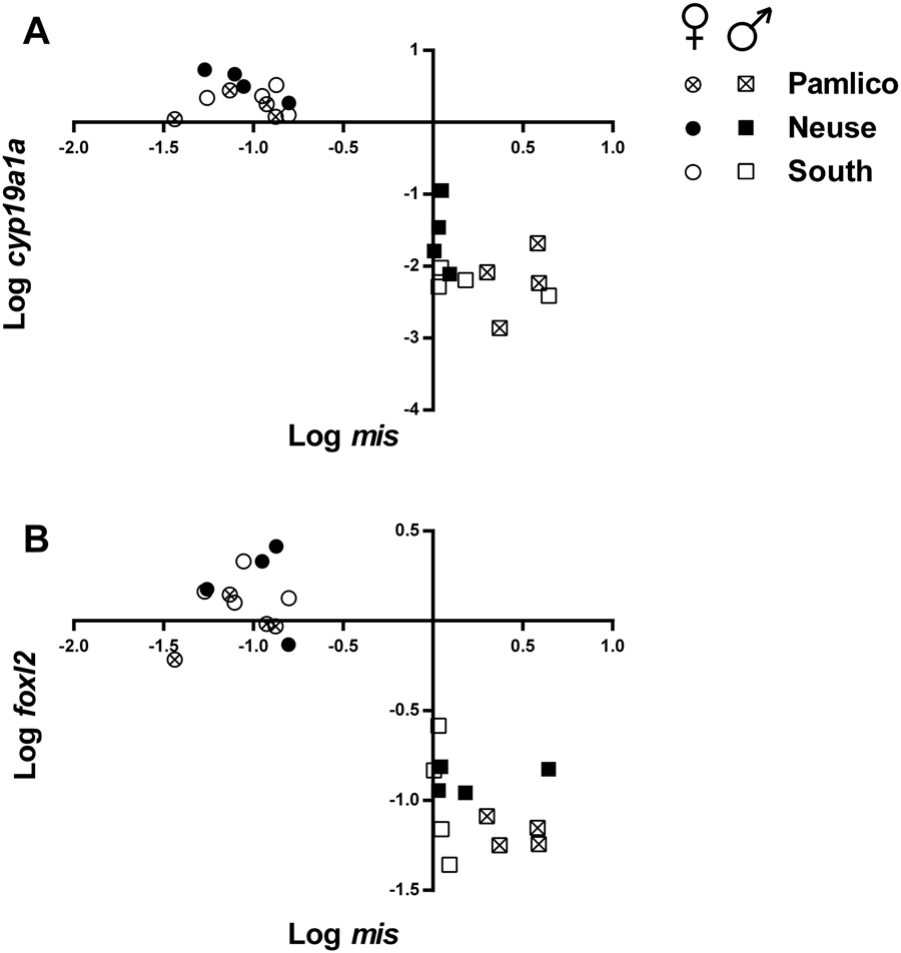
Mean log-transformed levels of gonadal mRNA of male (*mis*) and female (*foxl2* and *cyp19a1a*) biomarkers normalized to the reference gene *ef-1α* and plotted relative to each other from years 2014 to 2017. Shown above are dimorphic gonadal expression patterns for each marker (**A**) mean log *mis* as a function of mean log *foxl2* (**B**) mean log *mis* as a function of mean log *cyp19a1a*. Females are indicated by circles and males are indicated by squares. Samples from the Pamlico region are shown as circles/squares with an “x”, the Neuse region as black circles/squares, and the South region as white circles/squares.

**Figure 5:**
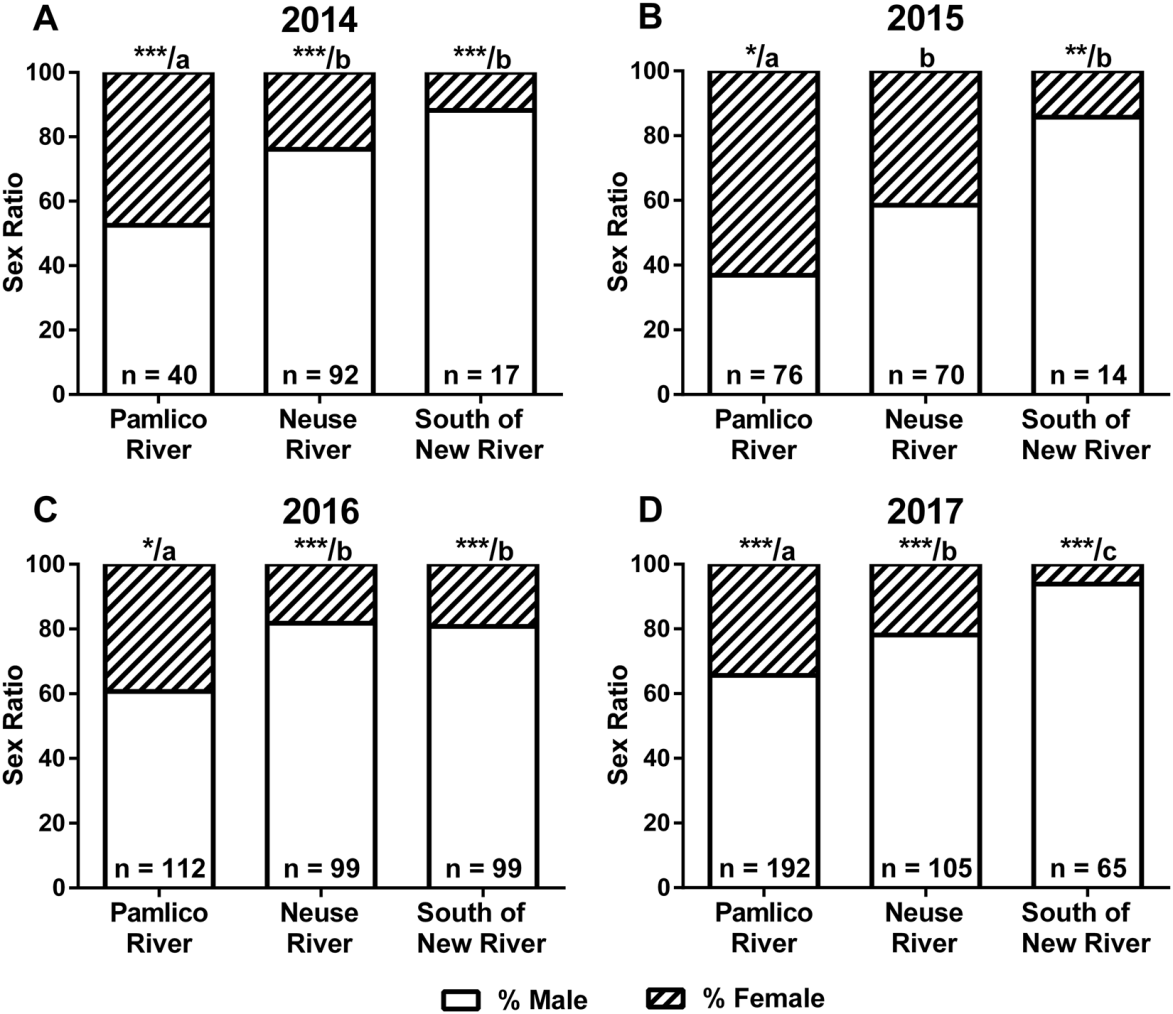
Sex ratios of juvenile southern flounder from different sampling regions Pamlico River (northern), Neuse River (intermediate), and South of the New River (southern) in NC, from 2014 to 2017. Sex ratios were determined by measuring gonadal gene expression of male (*mis*) and female (*cyp19a1a* and *foxl2*) via qPCR. Significant deviations (Chi-square) from a 50:50 sex ratio are noted as follows: (**A**) 2014 ***P < 0.0001, (**B**) 2015 *P = 0.02, **P = 0.007, (**C**) 2016 *P = 0.02, ***P < 0.0001, and (**D**) 2017 ***P < 0.0001. Differences between regions within each sampling year are denoted with different letters (Fisher’s Exact Test). All comparisons indicated significant differences except between the Neuse and South regions in 2014 to 2016 (in 2015 *P* = 0.071).

The sex of each individual fish was identified with gonadal biomarkers of *foxl2*, *cyp19a1a*, and *mis*^37^. All flounders were sexed first as individuals and then grouped by region. Mean gene expression levels for the female markers (*foxl2* and *cyp19a1a*) vs. the male marker (*mis*) for each region over four years are shown in Fig. 4. As previously shown, there is a clear upregulation of the female markers (*foxl2* and *cyp19a1a*) compared to the male marker (*mis*) in female fish and vice versa in males^37^. We anticipated 1:1 sex ratios in all habitats if there is no effect from the environment, so all sex ratios were compared to the expected 50% male. There were significant differences from 1:1 sex ratios across regions over all years of data collection for 10 out of 12 comparisons (Chi-square; Fig. 5). Preliminary data collected in 2012 (n = 55) and 2013 (n = 73), when samples were only collected from the Neuse River, produced sex ratios that were significantly biased toward males (88% males in 2012, 82% in 2013; all *P* < 0.0001, data not shown). In 2014, the percentages of fish classified as males were 52% in the Pamlico (P > 0.05; n = 40), 76% in the Neuse (*P* < 0.0001; n = 92), and 88% in South of the New River (*P* < 0.0001; n = 17). In 2015, the percentages of fish classified as males were 37% in the Pamlico (*P* < 0.02; n = 76), 59% in the Neuse (P > 0.05; n = 70), and 86% in South of the New River (*P* < 0.007; n = 14). In 2016, the percentages of fish classified as males were 61% in the Pamlico (*P* < 0.02; n = 112), 82% in the Neuse (*P* < 0.0001; n = 99), and 81% in South of the New River (*P* < 0.0001; n = 99). In 2017, 67%, 78%, and 94% of fish were males in the Pamlico (n = 186), Neuse (n = 105), and South of New River (n = 65) regions, respectively (all *P* < 0.0001). A Fisher’s Exact Test was used to compare differences between regions in each sampling year. All comparisons indicated significant differences except between the Neuse and South regions in 2014 to 2016 (in 2015 *P* = 0.071).

### Temperatures from Tank Study

**Figure 6:**
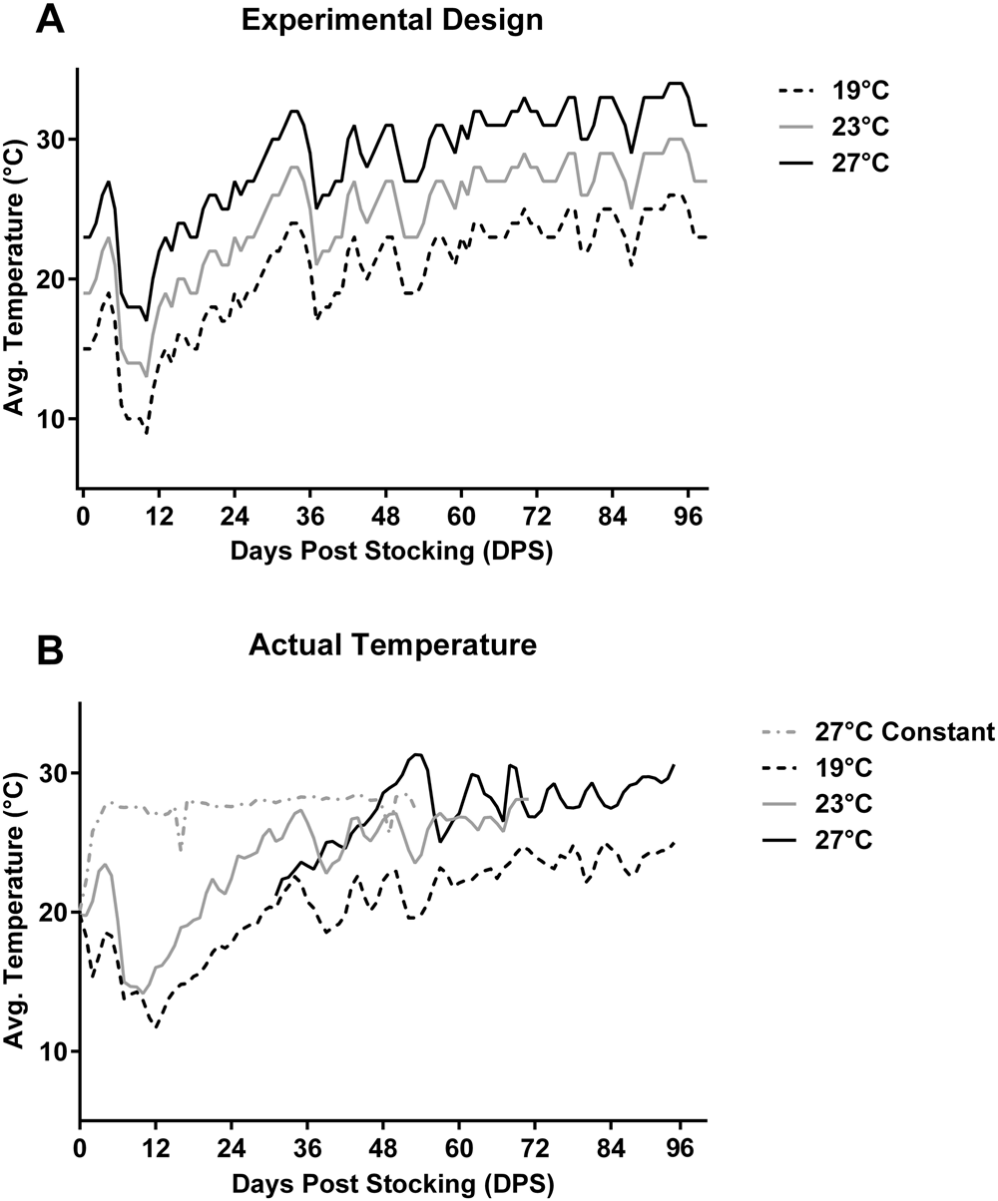
Temperature profiles from an experiment with juvenile southern flounder that were reared in different fluctuating temperature profiles that deviate by 4 °C throughout the period of sex determination. (**A**) The experimental design temperature profiles for 19 °C, 23 °C and 27 °C average temperature treatments. (**B**) The actual recorded daily average temperature (°C). Actual average temperatures of each system recorded during the sex determination window were 18.3 °C, 21.8 °C, and 26.8 °C respectively. Additionally, there was a 27 °C constant treatment with an actual average temperature of 27.1 °C.

To test if temperature alone could produce sex ratio differences consistent with variations in natural habitats, we reared southern flounder in different temperature regimes averaging 19 °C, 23 °C, and 27 °C (Fig. 6A). The 23 °C treatment was based on Swanquarter Bay, a site in the Pamlico region characterized by 50:50 sex ratios and where the recorded temperature in 2014 was on average 23.2 °C during the estimated period of sex determination. The 19 °C and 27 °C treatments reflected 4 °C deviations from the Swanquarter profile, a temperature difference that we found occurs between southern and northern habitats. As a positive control for sex reversal, there was a fourth system held at a constant 27 °C (28 °C produced 96% males^35^). Initial starting temperatures for the four systems were 19.8 °C, 18.8 °C, 20.0 °C and 20.1 °C and fish were held at these temperatures for one week before the experiment and temperature fluctuations were initiated. Actual average daily temperatures of each system during the sex determination window (i.e. when fish were <65 mm TL) were 18.3 °C, 21.8 °C, 26.8 °C, and 27.1 °C respectively (Fig. 6B). Over the entire study the average daily temperatures for each system were 20.5 °C, 23.5 °C, 27.5 °C, and 27.5 °C, for the 19 °C, 23 °C, 27 °C and constant 27 °C treatments respectively.

### Sex Ratios from Fluctuating Temperatures in Tanks

**Figure 7:**
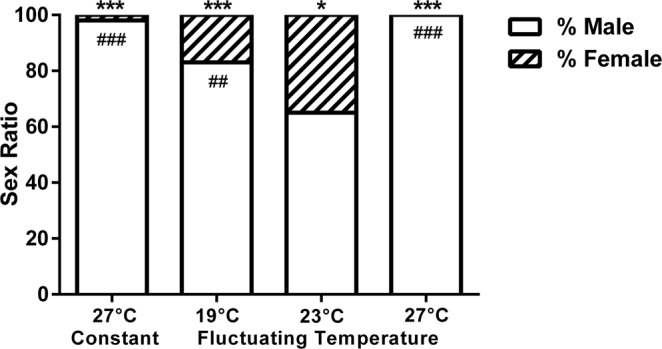
Sex ratios of southern flounder reared in different fluctuating temperature regimes mimicking natural temperature profiles from juvenile southern flounder nursery habitats, and of a group reared in constant 27 °C that served as a control for masculinizing flounder. Nominal average temperatures in the treatments were 19 °C, 23 °C, and 27 °C throughout sex determination and sex ratios were 83%, 65%, 100% male respectively. The 27 °C constant system produced 98% male. (n = 48 per treatment, Chi-square: *P = 0.04, ***P < 0.0001; all *P* represent significant deviations from a 50:50 sex ratio). The proportion of males was significantly higher for all other treatments relative to the 23 °C fluctuating group (Chi-square: ^##^*P* = 0.004 for 19 °C and ^###^*P* < 0.0001 for 27 °C fluctuating and 27 °C constant temperature groups; *P* represents significant deviations from the 65:35 male:female sex ratio). Sex ratios were determined by measuring gonadal gene expression of male (*mis*) and female (*cyp19a1a* and *foxl2*) markers via qPCR.

Sex ratios were calculated using gonadal biomarkers for *foxl2*, *cyp19a1a*, and *mis*. The 27 °C constant temperature system that served as a control for masculinizing flounder produced 98% males. The 19 °C, 23 °C, and 27 °C fluctuating temperature groups produced sex ratios that were 83%, 65% and 100% male, respectively (Fig. 7, n = 48 per treatment, Chi-square: 23 °C *P* = 0.04, all others *P* < 0.0001; all *P* represent significant deviations from a 50:50 sex ratio). Although the 23 °C fluctuating treatment was mildly male biased (65% male), it produced more females than any other treatment, followed by the 19 °C group (83% male). The 27 °C constant system produced only one female, and the 27 °C fluctuating system produced no females. All treatments produced male-biased sex ratios that were significantly different from the 23 °C fluctuating system (19 °C *P* = 0.004; 27 °C constant and fluctuating *P < *0.0001). There were no individuals whose sex was deemed undetermined from any treatment.

## Discussion

This is the first study to show consistent and significant geographic variation in juvenile sex ratios in southern flounder from productive nursery habitats. Importantly, those habitats producing the highest proportions of males also had consistently warmer water temperatures than those with lower proportions of males, as was seen in more southerly versus northerly NC nursery habitats.

As anticipated, temperature in nursery habitats varied annually. Temperatures recorded over an equivalent spring timeframe in all habitats were significantly higher in 2015 and 2017 compared to 2016. However, this disparity in temperature did not appear to directly correlate to differences in overall sex ratios observed in those years. The proportion of males was higher in the Pamlico and Neuse regions in 2016 despite recording a lower average annual temperature than either 2015 or 2017. Although all three regions produced a greater proportion of males in 2017, this was not the case for 2015, a year that produced a higher proportion of females in the Pamlico and Neuse regions. Temperature is a cue for spawning in southern flounder, with the cooler water temperatures of fall and winter initiating an emigration to offshore waters^24,25^. Variable temperatures during spawning could lead to interannual differences in sex ratios as it might lead to changes in the time of spawning and subsequently when larvae are transported back inshore to settle into nursery habitats, and ultimately what temperatures they are then exposed to as juveniles^43^.

Temperature also varied with latitude across sampled habitats, with the most northern sites in the Pamlico region producing the coolest temperatures, and sites South of the New River recording the warmest temperatures over all sampling years. Interestingly, during the period of sex determination (i.e., when flounder would be expected to be 35–65 mm TL), the Pamlico region averaged closest to 23 °C, which was the temperature found to produce the highest proportion of females in laboratory trials^35^. This region produced the greatest proportion of females each year, exhibiting close to 50:50 sex ratios in 2014 and was even slightly female-biased in 2015. Sex ratios were more variable in the Neuse River with male-biased ratios produced most years (up to 88% male) except for 2015 when the sex ratio was 59% male. The region South of the New River consistently produced male-biased sex ratios over all years (81% to 94% male).

These patterns of latitudinal variation in sex ratios in southern flounder have parallels in another well-studied species of fish that exhibits TSD. Some of the first studies to examine TSD in fish were conducted with the Atlantic silverside (*Menidia menidia*) and to date, effects of the environment on sex have been extensively studied in this species in the laboratory and the field^13^. Studies show a correlation between latitude and the degree of ESD observed in *M*. *menidia*, with ESD more predominant in northerly latitudes where the length of the growing season declines^43,44^. For southern flounder it appears that ESD is more prevalent at southerly latitudes (at least in NC), with the greatest masculinization occurring in southern habitats compared to those farther north. Spawning of southern flounder begins earlier at more northerly latitudes and progresses southward over time^24^. This suggests that flounder larvae would settle earlier into those northern environments like the Pamlico, compared to the southern habitats. Although the fish in these studies were not aged, regional differences in size are consistent with this pattern. The overall smaller body size of juvenile flounder in the southern region suggests that they could be younger, and thus would reach the critical sex-determining window later in the summer when water temperatures are much higher and more likely to promote male development, similar to the pattern found in *M*. *menidia*^13^. In conjunction with identifying phenotypic sex, precisely determining the age of YOY southern flounder both spatially and across the months of the spring season when fish settle into their nursery habitats could more accurately characterize the timing and impact of environment and temperature on sex determination.

Another possibility for the smaller size of the flounder collected South of the New River is that the fish in this region might grow more slowly, thereby extending the window over which they are sensitive to their environment. The growth rates observed in NC populations of southern flounder can vary considerably, from 0.35 to 1.5 mm per day and can vary regionally^28^. The differential growth observed among juveniles across regions could be due to food size and availability, although southern flounder that spawned earlier were not shown to be larger or faster growing than those spawned later in the season^28^. Female southern flounder have been shown to grow faster and larger than males, but typically this sexually dimorphic growth does not begin until larger body sizes (~200 mm TL^28^). Therefore, sex differences in growth should not account for size disparities of smaller juveniles. Surprisingly, flounder from the southern region had higher HSI but smaller body sizes. We measured HSI because fish store energy in the form of glycogen and fat in their liver tissue that can be mobilized in times of stress^45^. This index often correlates to somatic growth as shown in previous laboratory studies of southern flounder^42^. It seems unlikely therefore that lack of food availability is responsible for the smaller body size of fish in southern regions. However, this was a field study and other factors such as temperature or diet composition could account for the lack of concordance between HSI and body size.

Age-0 southern flounder in South Carolina (SC) have been shown to have bimodal distributions in length at 50 and 140 mm TL in June^46^. This phenomenon is similar to what we observed over multiple years South of the New River and could suggest that the timing of spawning and juvenile growth in this region is more comparable to that of SC populations. For example, in 2016 we captured two size groups of southern flounder and they were on average 56.1 mm TL and 145.6 mm TL. Although we did capture larger age-0 flounder in the Pamlico and Neuse regions, there was not the clear presence of two distinct size groupings as observed in the south. Previous studies showed that bimodal length distributions in age-0 NC southern flounder were independent of sex, generally occurred around 75 to 100 mm TL, and were likely due to a change to a piscivorous diet^28^. Interestingly, female flounder were more prevalent in the larger size group of fish we captured South of the New River, suggesting these fish determined sex at a time when conditions were more optimal for female development.

Additionally, these studies show that when southern flounder are reared in fluctuating temperatures in the laboratory intended to mirror those of natural temperature regimes, the sex ratio differences observed among these natural habitats are recapitulated. Temperature profiles across different nursery habitats exhibited similar patterns of fluctuations, although between any two habitats temperature could differ in mean recordings more than 4 °C. In addition, a northern nursery habitat in the Pamlico Sound (Swanquarter Bay) that produced 1:1 sex ratios had an average temperature of 23.2 °C in 2014 during the sex determination period. This is consistent with the observed optimal temperature for production of females of 23 °C that yields 1:1 sex ratios in the laboratory^35^. In the current study, when the temperature profile of this habitat was reproduced in a recirculating system to obtain temperature fluctuations that similarly averaged 23 °C during sex determination, this regime produced the greatest proportion of female flounder (35% female), which was significantly higher than those of the 19 °C (17% female) or 27 °C (0% female) treatments (Fig. 7). Although the 23 °C fluctuating treatment yielded the greatest proportion of females, it was still mildly male biased at 65% male based on an expected 1:1 sex ratio. This ratio is comparable to what was observed for the northern Pamlico region for 2016 and 2017, where sex ratios were 61% and 67% respectively, while other sampling habitats farther south produced a greater proportion of males both years (82% and 94% male). The 27 °C fluctuating treatment that produced 100% males was representative of the southern sampling habitats, South of the New River, which also displayed significantly male-biased sex ratios in field collections. Although previous field sampling did not include a habitat comparable to the 19 °C treatment, it could be representative of nursery environments farther north of our study areas towards the Albemarle Sound, a region in NC that juvenile southern flounder are also known to inhabit^27^, or possibly even habitats farther north as projections of shifting habitats and species distributions northward due to climate change have been predicted for the North American coasts^9^. The results of our study, conducted with other physical parameters (i.e. salinity, oxygen, light, etc.) maintained similarly across treatments, suggests that temperature may be the predominant factor that is influencing sex determination in natural habitats.

Warm temperatures have been strongly correlated to masculinized sex ratios in southern flounder in fish reared in tanks (constant temperatures^35^, fluctuating temperatures; current study) and now in natural populations. Exposure to temperature fluctuations has been shown to impact sex ratios in fish and turtles that exhibit TSD/ESD (*M*. *menidia*^13^; *Carassius auratus*^47^; *Pimephales promelas*^48^; *Trachemys scripta*^49^). In the case of sea turtles, cool temperatures tend to produce males and warm temperatures produce more females, so global warming could feminize sea turtle populations^21^. A recent study on sex ratios of sea turtles near the Great Barrier Reef showed variation in sex ratios across regions, with mildly female-biased ratios in cooler, southern beaches and extreme female-biased sex ratios in warmer more northern nesting sites^22^. This latitudinal variation in sex ratios of natural turtle populations shows a response due to TSD in a warming environment similar to what we observed across a temperature cline in southern flounder. This phenomenon may not be limited to turtles and southern flounder and should be investigated for other species that exhibit TSD.

Overall, our findings indicate that there is consistent variation in the water temperatures across nursery habitats in NC that are inhabited by juvenile southern flounder during the critical period of development for ESD/TSD. These temperature differences are associated with variation in juvenile sex ratios across regions. Warmer nursery habitats in southern NC produced significantly more male southern flounder compared to those environments farther north. We were able to reprise these differences under controlled laboratory conditions, providing further evidence that water temperature is causally responsible for the sex ratio differences observed in nursery habitats. These studies provide evidence of TSD affecting a key demographic parameter in wild populations of an important fishery species. With a fishery dependent on females and global ocean temperatures projected to significantly increase, these temperature effects on sex ratios could be a significant concern for wild flounder stocks and potentially other species that exhibit TSD.

## Methods

### Sampling Wild Juvenile Southern Flounder

Juvenile southern flounder were collected with the assistance of North Carolina Division of Marine Fisheries (NCDMF) biologists during the routine juvenile monitoring Program 120 (P120), the North Carolina Estuarine Trawl Survey. This survey samples shallow, upper estuary locations typically in May and June using an otter trawl with a 3.2 m headrope, 6.4 mm bar mesh wings and body, and 3.2 mm bar mesh cod end. At each station, the trawl is towed for 1 min at 1.1 m/s covering ~69 m. We supplemented NCDMF southern flounder collections with our own sampling efforts using similar gear (otter trawl) or using a 2 m wide beam trawl depending on the habitat. Sampling was focused on collecting YOY age-0 southern flounder 50 mm TL and larger (this is the body size at which sex-specific molecular biomarkers begin to be expressed). These fish were collected over a wide spatial area to test the null hypothesis that there is no effect of region on sex ratio. Sampling regions included: (1) the Pamlico region, the most northern of our sampling sites including the Pamlico River and tributaries, (2) the Neuse River; an intermediate region including three sites, and (3) South of the New River, the most southern region, primarily sampled from Mill Creek and then supplemented with fish from two other nearby creeks (Fig. 1). We aimed to capture a minimum of 50 flounder from each of the three study regions in each of the sampling years, 2014 to 2017. Samples were also obtained in 2012 and 2013, but only from the Neuse region for these years. Fish were kept alive with a battery-operated aerator until sampling could be completed. Immediately prior to dissection, the fish were euthanized using a lethal dose of buffered MS-222 (Pentair Aquatic Eco-Systems, Apopka, FL), and then weighed (g), and measured for total length (mm TL). Whole liver tissue was removed and weighed to obtain the hepatosomatic index (HSI; (liver weight/total body weight) × 100). Gonadal tissue was dissected, placed in 0.5 mL of RNAlater (Invitrogen, Waltham, MA) overnight at 4 °C and then stored at −20 °C until RNA extractions and later analysis for expression of sex-specific biomarkers via quantitative real-time PCR (qPCR) to determine sex ratios.

### Habitat Water Temperature

Water temperature was recorded every 2 hours using iButton temperature loggers (DS1922L −40 to 85 °C Thermochron, iButtonLink Technology, Whitewater, WI) enclosed in a waterproof housing. Temperature loggers were deployed in sampling locations and juvenile southern flounder habitats throughout the spring and summer (typically late March to early July) from 2014 to 2017 (Fig. 1, also see Supplemental Table 1 for GPS coordinates). Data were averaged over each 24-hour period to generate an average daily measurement for each habitat. Temperature, dissolved oxygen, and salinity measurements were also taken at the time of juvenile fish sampling.

### Fluctuating Temperature Tank Study

Southern flounder were spawned at the University of North Carolina at Wilmington, Center for Marine Science (UNCW-CMS) in Wilmington, NC, and then reared at North Carolina State University (NCSU) in Raleigh, NC. Wild-caught southern flounder were maintained at UNCW-CMS and eggs and sperm were collected by strip-spawning from multiple broodstock and pooled for *in vitro* fertilization^50^. Fertilized eggs were transported to the NCSU Lake Wheeler laboratory in Raleigh, NC and larvae were reared in a 3000-L recirculating artificial seawater system until 60 days post hatch (dph). Larvae were fed a diet of live feed (rotifers and artemia) and gradually weaned onto a diet of high-protein dry feed (Reed Mariculture, Campbell, CA) that was continued through metamorphosis. All procedures and research were approved and performed in accordance with the relevant guidelines and regulations by the Institutional Animal Care and Use Committees at North Carolina State University and the University of North Carolina at Wilmington.

Post-metamorphic flounder at an average size of 24.6 ± 0.8 mm total length (TL) were stocked in triplicate in 100-L round fiberglass tanks on three separate recirculating aquaculture systems at a density of 250 fish/m^2^ for fluctuating temperature systems and at the same density in duplicate 340-L tanks in another system for the 27 °C constant control. All tanks were gray in color. Salinity started at ~29 ppt and was gradually decreased to and maintained at 5 ppt. Photoperiod was maintained on a 12 L:12D schedule using artificial lighting, all conditions previously shown to produce 1:1 sex ratios of flounder^37,51^. Fish were fed a commercial diet 3–5 times daily and any unconsumed food was siphoned out of tanks (Reed Mariculture, Campbell, CA; Otohime, range of sizes, ~ 51% crude protein, 11% crude fat). Temperatures were controlled to produce natural fluctuations in temperatures similar to what we observed in nursery habitats (rising over the course of the spring season) but with average daily temperature deviating by 4 °C from the control group, i.e. average temperatures of 19 °C, 23 °C, and 27 °C over the course of the experiment (Fig. 6A). The 23 °C temperature profile is based on measurements from the Swanquarter bay location during the estimated sex determining window in 2014 (Latitude: 35.385, Longitude: −76.312). This location in the Pamlico region produced 50:50 sex ratios that season and the average recorded temperature was 23.2 °C. The period of sex determination was estimated using daily growth rate calculations from fish sampled at two time points and then extrapolated backwards to estimate when fish would have been 35 mm (the beginning of the sex determination window). Additionally, when the average daily temperature is considered across all nursery habitats, the greatest mean difference recorded between all locations was near 4 °C, so treatments were varied by this amount. Actual water temperatures in the systems were recorded every 2 hours using iButton temperature loggers (DS1922L − 40 to 85 °C Thermochron, iButtonLink Technology, Whitewater, WI) enclosed in a waterproof housing, and secured to a standpipe in one of the tanks on each treatment system. There were two dates, days 16 and 49 post-stocking, where the heater in the 27 °C constant system faltered so the temperature dropped to an average of 24.4 °C on day 16 and 25.6 °C on day 49, but these incidents did not substantially impact the average water temperature as it was calculated to be 27.5 °C over the course of the experiment.

Temperature was maintained and adjusted daily to fit the assigned profile through a combination of submersible heaters and ¼ hp drop-in titanium chillers (CY-3-CWCT, Aqualogic Inc., San Diego, CA) for each of the three systems. The 27 °C fluctuating system failed 28 days into the study and was restarted three days later with flounder transferred from the 19 °C system as they were still on average 31.3 ± 1.3 mm TL, prior to the start of the window of sex determination (35–65 mm TL). Survival was not different among temperature treatments. Animals were grown out to an average of 102 ± 0.6 mm TL. At the termination of the experiment and immediately prior to dissection, the fish were euthanized using a lethal dose of buffered MS-222, and then weighed (g), and measured for length (mm TL). Gonadal tissue was dissected, placed in 0.5 mL of RNAlater overnight at 4 °C and then stored at −20 °C until later analysis for expression of sex-specific biomarkers.

### RNA Isolation and cDNA Synthesis

Total RNA was extracted from all gonadal tissue with 1 mL of TRI-reagent and 6 µl of Polyacryl carrier (Molecular Research Center, Cincinnati, OH) using standard methods from the manufacturer, and pellets were resuspended in 20 µl of nuclease-free water. RNA was quantified by absorbance OD 260:280 ratio using a Nanodrop 1000 spectrophotometer (Thermo Fisher Scientific, Waltham, MA). RNA quality was assessed when there was adequate sample volume by presence of 18S and 28S ribosomal RNA bands using gel electrophoresis. All RNA was DNase treated (Turbo DNA-free, Invitrogen, Waltham, MA) to eliminate genomic DNA, then re-quantified and diluted to 100 ng/µl unless concentrations were already below 100 ng/µl. The size of the gonadal tissue samples varied based on the age and size of the fish so the quantity of RNA extracted varied among individuals. Therefore, a range of 0.4 to 1.0 µg of total RNA was used to synthesize cDNA via reverse transcription (High Capacity cDNA Synthesis Kit, Applied Biosystems, Waltham, MA).

### Determining Sex Ratios by Real-time Quantitative PCR

Expression of gonadal biomarkers were used to sex individual southern flounder using qPCR as previously validated in this species^37,52^. Müllerian-inhibiting substance (*mis*; also known as anti-müllerian hormone, *amh*) was used as a marker for male development, Forkhead transcription factor L2 (*foxl2*) and P450 aromatase (*cyp19a1a*) were used as female markers, and elongation factor-1 alpha (*ef-1α* or *eef1α*) was used as a reference gene. Gene specific primers were used to measure the expression of these markers (GenBank accession numbers: *foxl2*: KF534720, *mis*: KF534719, *cyp19a1a*: AY902192, and *ef-1α*: AY884199). Sequences of primers used for qPCR are as follows: *foxl2* forward primer (FP): GTCCCCGCCCAAGTACCT, *foxl2* reverse primer (RP): GGCCGAGCGACCATGAG, *mis* FP: CTGCCGAGGCTCTTGCA, *mis* RP: CAGGACGGCATGGTTGATG, *cyp19a1a* FP: GGAGCCACACAGACAGGAGAA, *cyp19a1a* RP: GGCCCCAAACCCAGACA, *ef-1α* FP: CGAGAAAGAAGCTGCCGAGAT, *ef-1α* RP: CGCTCGGCCTTCAGTTTGT. qPCR analyses were performed on a BioRad CFX384 instrument, using Brilliant II SYBR Green qPCR master mix (Agilent Technologies, Santa Clara, CA), using 1.5 µM primers, and 2 µL of 1:6 diluted cDNA in a total reaction volume of 10 µL. The qPCR cycling parameters were 95 °C for 10 min followed by 40 cycles of 95 °C for 30 sec and 60 °C for 1 min. A final melt curve step was performed to verify a primer specificity for each gene with the presence of a single peak. The absence of genomic DNA contamination was confirmed using water (No Template Control; NTC) and DNase-treated RNA (No-Amplification Control; NAC) as negative control templates setup during cDNA synthesis. Cycle threshold (Ct) values for samples were transformed using a standard curve of serially diluted pooled cDNA versus Ct values (R^2^ = 0.96–0.99). Samples were then normalized to reflect the amount of template cDNA per ng total RNA loaded into each reaction (cDNA/ng total RNA). Additionally, we quantified expression levels of *ef-1α* RNA as a reference gene, as the expression of *ef-1α* had previously been shown not to vary significantly across tissues (validated as a reference gene for southern flounder gonad mRNA in^52^). As such all data were normalized using the two methods, both to the expression of *ef-1α* and the cDNA/ng total RNA, and then compared. Both normalization methods produced similar results.

All samples were analyzed for expression of *foxl2*, *cyp19a1a*, and *mis* using qPCR. Ratios of the levels of the three genes were established in each individual sample. Samples with relative high expression of *foxl2* and *cyp19a1a* and with low *mis* expression were identified as female, samples with relative high expression of *mis* and with low levels of *cyp19a1a* and *foxl2* were identified as male, and those samples with low or uncharacteristic expression of all sex biomarkers were classified as undetermined^37^.

### Statistical analyses

All sex ratios were analyzed by region or treatment and compared against a predicted 1:1 sex ratio using Chi-square goodness-of-fit tests. Significance between regions within a sampling year was assessed with a Fisher’s Exact Test. HSI and annual temperature were analyzed using a two-way analysis of variance, ANOVA, with multiple comparisons (region by year and across years) followed by Tukey’s honest significant difference (HSD) post-hoc test for multiple comparisons within years (HSI) and across years (annual temperature). All analyses were performed using JMP (Pro v13, SAS Institute, Cary, NC) and/or GraphPad Prism 6 (GraphPad, La Jolla, CA). The threshold set for statistical significance for all analyses was *P* < 0.05. Data are expressed as the mean ± SEM.

The datasets generated during and/or analyzed during the current study are available from the corresponding author on reasonable request.

## Supplementary information

Supplemental data
