# Supplementary material for: Warmer waters masculinize wild populations of a fish with temperature-dependent sex determination
Source: Sci Rep. 2019 Apr 25;9:6527. doi: 10.1038/s41598-019-42944-x (PMC6483984; doi:10.1038/s41598-019-42944-x)
Supplement: Supplementary file 1 — Supplemental data [file 41598_2019_42944_MOESM1_ESM.pdf]

Warmer waters masculinize wild populations of a fish with temperature-dependent sex determination

Honeycutt, J.L.[1], Deck, C.A.[1], Miller, S.C.[2], Severance, M.E.[1], Atkins, E.B.[1], Luckenbach, J.A.[3], Buckel, J.A.[2], Daniels, H.V.[2], Rice, J.A.[2], Borski, R.J.[1,*], Godwin, J.[1,*]

[1]North Carolina State University, Department of Biological Sciences, Raleigh, NC 27695, USA
[2]North Carolina State University, Department of Applied Ecology, Raleigh, NC 27695, USA
[3]Environmental and Fisheries Sciences Division, Northwest Fisheries Science Center, National Marine Fisheries Service, National Oceanic and Atmospheric Administration, 2725 Montlake Blvd E, Seattle, WA 98112, USA

[*]Correspondence: godwin@ncsu.edu or russell_borski@ncsu.edu

SUPPLEMENTAL DATA

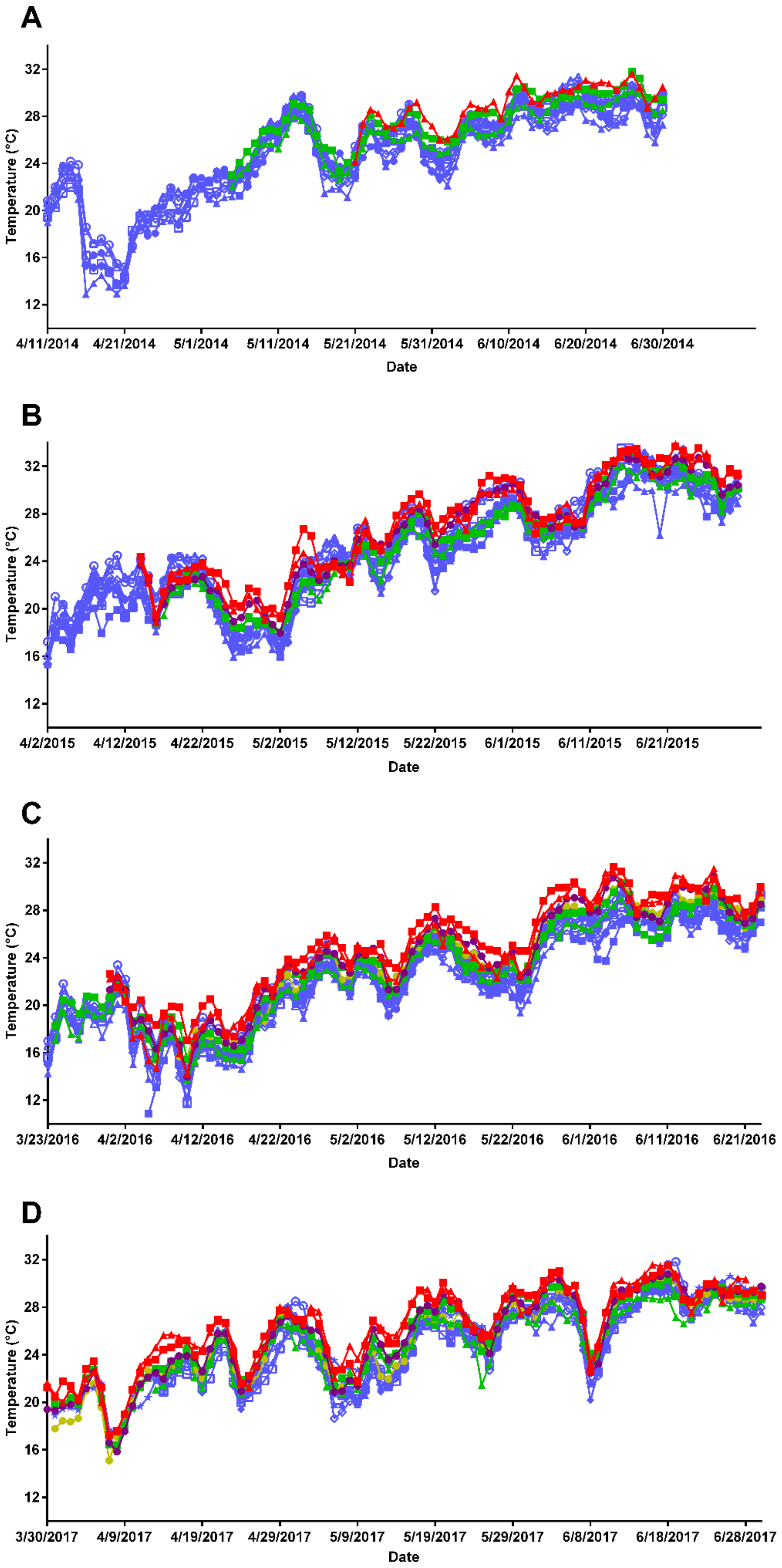

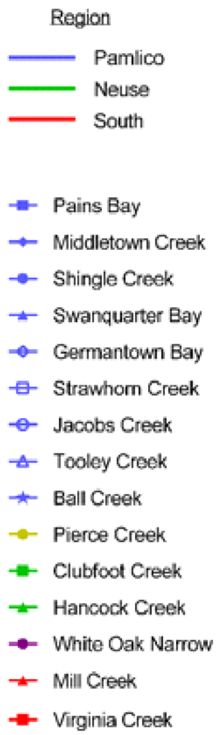

Supplemental Figure 1. Average daily water temperature from juvenile southern flounder habitats over four years, (**A**) 2014 (April 11 to June 30), (**B**) 2015 (April 2 to June 30), (**C**) 2016 (March 23 to June 30) and (**D**) 2017 (March 30 to June 30). Data were recorded with iButton loggers every 2 hours and temperature readings were averaged over a 24-hour period.

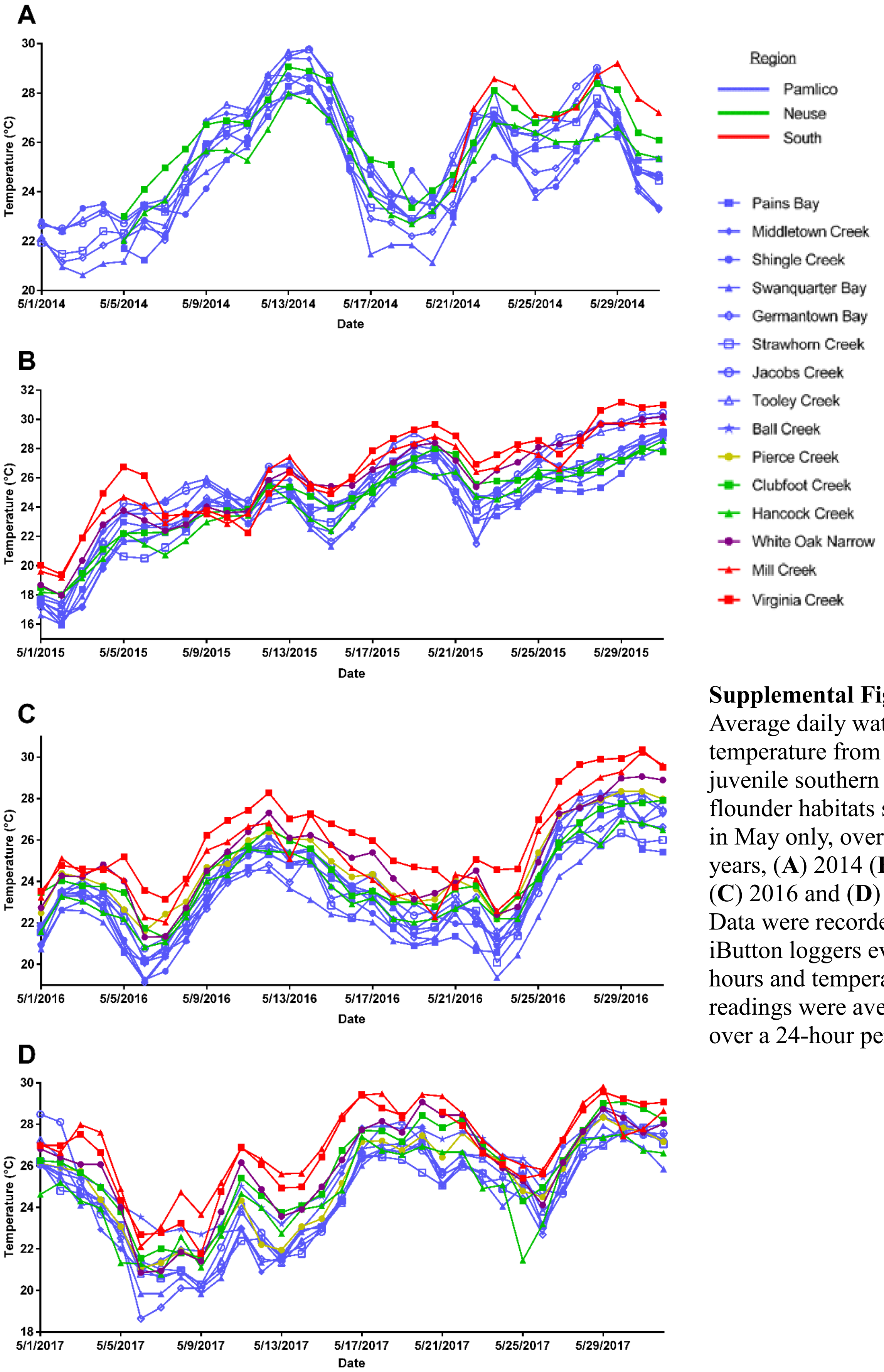

Supplemental Figure 2. Average daily water temperature from juvenile southern flounder habitats shown in May only, over four years, (**A**) 2014 (**B**) 2015 (**C**) 2016 and (**D**) 2017. Data were recorded with iButton loggers every 2 hours and temperature readings were averaged over a 24-hour period.

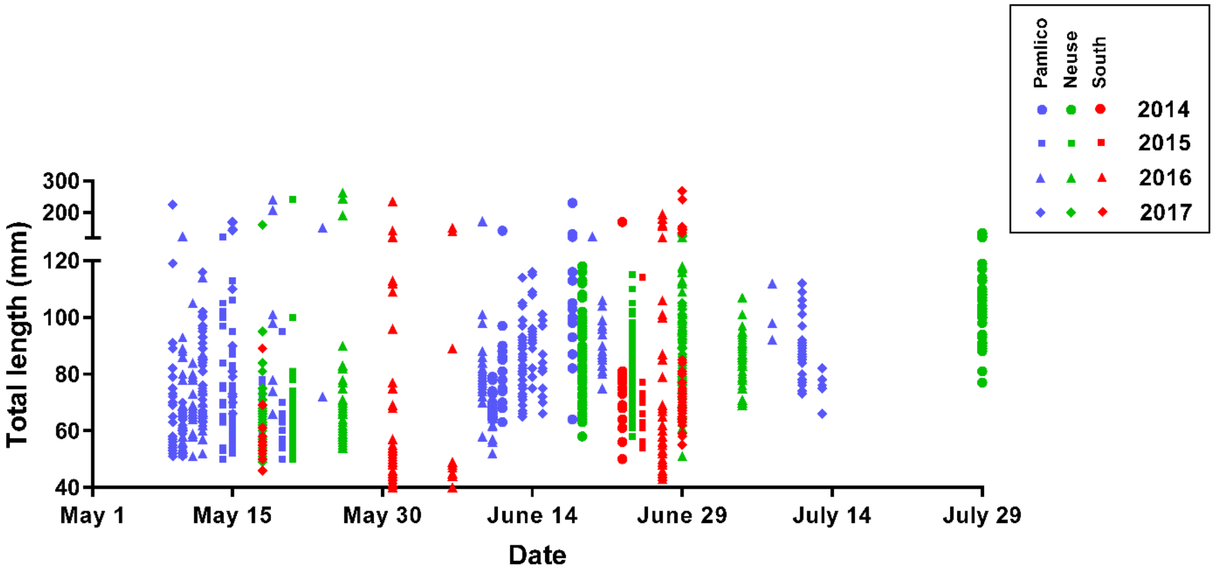

Supplemental Figure 3. Total length (mm TL) of juvenile southern flounder over time from collections in May to July over four years, 2014 (circles), 2015 (squares), 2016 (triangles), and 2017 (diamonds), in three different regions: Pamlico (north, blue), Neuse (intermediate, green), and South (red).

Supplemental Table S1. GPS Coordinates for temperature probes deployed in juvenile southern flounder habitats in North Carolina from 2014 to 2017.

Location	NCDMF Station #	Region	Water Body	Latitude	Longitude
Middletown Creek	FC3	Pamlico	Far Creek	35.474	-76.008
Pains Bay	LSR5	Pamlico	Long Shoal River	35.598	-75.818
Germantown Bay	SB5	Pamlico	Germantown Bay	35.422	-76.446
Swanquarter Bay	SQB3	Pamlico	Swanquarter Bay	35.385	-76.312
Shingle Creek	SQB1	Pamlico	Swanquarter Bay	35.414	-76.357
Strawhorn Creek	PAR16	Pamlico	South Creek	35.340	-76.644
Tooley Creek	PAR27	Pamlico	South Creek	35.347	-76.749
Jacobs Creek	PAR31	Pamlico	South Creek	35.336	-76.765
Ball Creek	CS2	Pamlico	Bay River	35.148	-76.636
Pierce Creek	F3N	Pamlico/Neuse	McCotter Bay	35.043	-76.666
Clubfoot Creek	H2	Neuse River	Neuse River	34.865	-76.762
Hancock Creek	NR11	Neuse River	Neuse River	34.935	-76.852
White Oak Narrow	CC1	White Oak River	White Oak River	34.709	-77.076
Virginia Creek	VC1	South of the New River	Virginia Creek	34.433	-77.606
Mill Creek	SSI1	South of the New River	Mill Creek	34.518	-77.424
